# Bioink Formulation and Machine Learning-Empowered Bioprinting Optimization

**DOI:** 10.3389/fbioe.2022.913579

**Published:** 2022-06-13

**Authors:** Sebastian Freeman, Stefano Calabro, Roma Williams, Sha Jin, Kaiming Ye

**Affiliations:** ^1^ Department of Biomedical Engineering, Binghamton University, Binghamton, NY, United States; ^2^ Center of Biomanufacturing for Regenerative Medicine, Binghamton University, State University of New York (SUNY), Binghamton, NY, United States; ^3^ Department of Biomedical Engineering, University of Miami, Coral Gables, FL, United States

**Keywords:** bioprinting, bioink, bioink formation, biomaterials, biofabrication, additive biomanufacturing, machine learning, tissue engineering

## Abstract

Bioprinting enables the fabrication of complex, heterogeneous tissues through robotically-controlled placement of cells and biomaterials. It has been rapidly developing into a powerful and versatile tool for tissue engineering. Recent advances in bioprinting modalities and biofabrication strategies as well as new materials and chemistries have led to improved mimicry and development of physiologically relevant tissue architectures constituted with multiple cell types and heterogeneous spatial material properties. Machine learning (ML) has been applied to accelerate these processes. It is a new paradigm for bioprinting. In this review, we explore current trends in bioink formulation and how ML has been used to accelerate optimization and enable real-time error detection as well as to reduce the iterative steps necessary for bioink formulation. We examined how rheometric properties, including shear storage, loss moduli, viscosity, shear-thinning property of biomaterials affect the printability of a bioink. Furthermore, we scrutinized the interplays between yield shear stress and the printability of a bioink. Moreover, we systematically surveyed the application of ML in precision in situ surgical site bioprinting, closed-loop AI printing, and post-printing optimization.

## Introduction

3D bioprinting has the potential to shift the paradigm in healthcare and biomedical research. Thousands of patients die every year waiting on organ donor lists in the United States (Morris, 2018). 3D bioprinting promises to alleviate this lack of suitable organs for transplant by fabricating patient-specific organs. It offers new tools for studying cell biology in more *in vivo*-like environments. Cells cultured in a 3D environment behave more closely to those *in vivo* (Habanjar et al., 2021; Jian et al., 2021). It has the potential of becoming a new standard for *in vitro* study (Li and Kilian, 2015; Caliari and Burdick, 2016; Smithmyer et al., 2019; Mills et al., 2020). Advances in 3D bioprinting makes it easier for researchers to create and study more complex cell and tissue models.

Most bioprinting apply additive manufacturing principles through the sequential and controlled spatiotemporal deposition of cells and biocompatible materials, which when mixed, are collectively referred to as bioinks. A variety of bioinks have been developed. However, to date, no single combination of bioprinter/bioink excels in all applications, which is why the field is still highly experimental. Innovation is very important to further advance this field. There are also no set criteria or standards for bioprinters or bioinks, which makes it difficult for researchers to decide what kind of bioprinter and bioink they need for their specific application. The number of commercially available bioprinters and bioinks has dramatically increased over the years, but it is likely that some research groups will continue to rely on custom setups and custom bioink formulations.

The development of bioinks for 3D bioprinting is a highly active research field. The ideal bioink possesses all the qualities necessary for successful bioprinting and to promote cell viability. It should also provide a suitable environment that supports cell proliferation, cell differentiation, and promote *in vivo* cellular behavior after printing. Macromolecular hydrophilic polymer hydrogels are commonly used as biomaterials for bioinks, as they can mimic extracellular matrix (ECM) environment. Indeed, many native ECM biopolymers have been used as bioinks.

The material requirement for pre- and post-printing can be drastically different. There are many examples of bioinks that demonstrate superb pre-printing qualities but poor post-printing qualities, specifically in terms of providing the necessary environment for proper cell and tissue development. Printed structures using sodium alginate (Zhang et al., 2013; Tabriz et al., 2015; Kang et al., 2016; Snyder et al., 2016), Pluronic F-127, (Wu et al., 2011; Ribeiro et al., 2018), or gelatin (He et al., 2016; Wang et al., 2019) have excellent structural integrity and high shape fidelity, enabling the printing of straight, consistent line filaments. Sodium alginate (Jia et al., 2014; Rastogi and Kandasubramanian, 2019; Bonani et al., 2020; Jiang et al., 2020; Gonzalez-Fernandez et al., 2021; Wu et al., 2021) and Pluronic F-127 (Atala and Yoo, 2015; Panwar and Tan, 2016), however, do not have cell binding sites for attachment without further modification. Gelatin contains binding sites for cell attachment (Davidenko et al., 2016) but will transition back to a liquid solution at 37°C, thus requiring a modification to convert it into a more permanent form (Tan et al., 2020). In cases where post-print crosslinking is required for long-term shape fidelity and mechanical performance, highly crosslinked hydrogels result in reduced oxygen and nutrient diffusion rates that are insufficient for encapsulated cell survival. In particular, one study found that average local strain surrounding cells in a polyethylene glycol (PEG) hydrogel increases in gels with higher degrees of crosslinking (Bryant et al., 2004). Results showed that due to increased crosslinking density, reductions in cellular DNA content, proteoglycan synthesis, and proliferation are evident. Furthermore, it has been found that photo-induced free radicals are a source of cytotoxicity toward human mesenchymal stem cells in photocrosslinked GelMA (O’Connell et al., 2018). Higher degrees of UV light-induced crosslinking resulted in decreased cellular metabolic activity. Although new chemistries and polymers are being studied to improve the available materials for bioprinting, a recent systematic review of bioinks up to 2017 revealed a divergence in bioink trends with more publication reporting the use of natural-material over synthetic-based bioinks, as well as an increasing trend of multicomponent bioink blends (Tarassoli et al., 2021).

We herein review the recent trends in bioprinting and bioink development and highlight new approaches being used to overcome limitations related to the printing of soft materials and the improvement of accuracy and resolution. Finally, we outline the strategies for streamlining the optimization of bioprinter-mediated biofabrication and current impeding limitations and future trends.

## Printability of Bioinks

**FIGURE 1:**
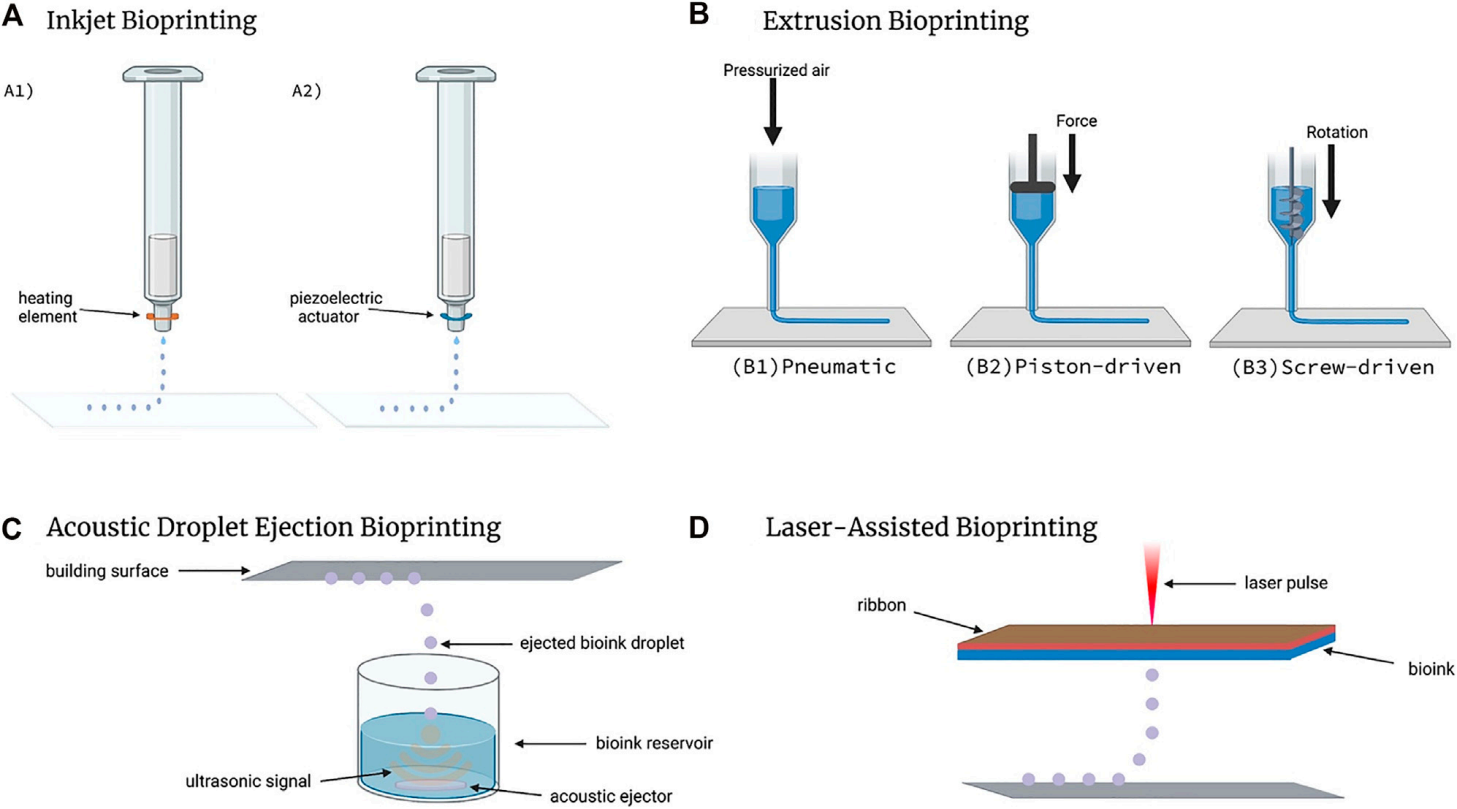
Types of Bioprinting. **(A)** Inkjet bioprinters create droplets through the use of A1) a heating element or A2) a piezoelectric actuator. **(B)** Extrusion bioprinters come in three unique configurations: B1) pneumatic, B2) piston-driven, and B3) screw-driven. **(C)** Acoustic Droplet Ejection bioprinting utilizes ultrasonic signals to eject bioink droplets onto the building surface. **(D)** Laser-assisted bioprinters deposit bioink droplets onto the building surface with each laser pulse. Created with BioRender.com.

With respect to bioinks, the term “printability” describes how successfully the bioink can be used to create the desired bioprinted structure, and is quantified by measuring the difference of the desired structure to the printed structure (Fu et al., 2021). The printability of a bioink is also dependent on the bioprinting modality and processing parameters. A printable bioink for one style of bioprinter may not be printable for another kind of bioprinter. For instance, the different bioprinting techniques that have been developed to realize various tissue manufacturing can be divided into three main categories: extrusion-based bioprinting, droplet-based bioprinting, and laser-based bioprinting. Extrusion-based bioprinting (EBB) is the most commonly used 3D biofabrication approach and is the leading manufacturing technique employed by tissue engineers to date (Gillispie et al., 2020). This modality is achieved by pushing bioink through a syringe and nozzle either mechanically or pneumatically. Droplet-based 3D bioprinting (DBB) can be described as the deposition of discrete, individual droplets that can be stacked and arranged into 3D structures. This printing modality can be further divided into three subcategories: inkjet bioprinting, acoustic-droplet-ejection bioprinting, and micro-valve bioprinting (Gudapati et al., 2016). Laser-based bioprinting, commonly referred to as laser-assisted bioprinting, uses lasers as its energy source to deposit material on the printing surface. Laser bioprinting modalities use nanosecond lasers with UV or near-UV wavelengths to irradiate a ribbon coated with liquid bioink, causing the biomaterial to evaporate onto a receiving substrate in droplet form (Li et al., 2016). The differences in functionality between each printing type, which can be seen in Figure 1, is what can lead to a bioink being printable in one style and not in another.

On-going research is connecting qualitative traits printed constructs to the material properties of the bioinks. Qualitative traits include printed line smoothness, shape fidelit or how close the printed shape approximates the original 3D model, its structural integrity, namely the ability to support its own weight, maintain its printed shape, printing accuracy and homogeneity of suspended cells. Below, we examine several physical and quantitatively measurable qualities that have been linked to the printability of different bioinks.

### Shear Storage, Loss Moduli, and Loss Tangent

**FIGURE 2:**
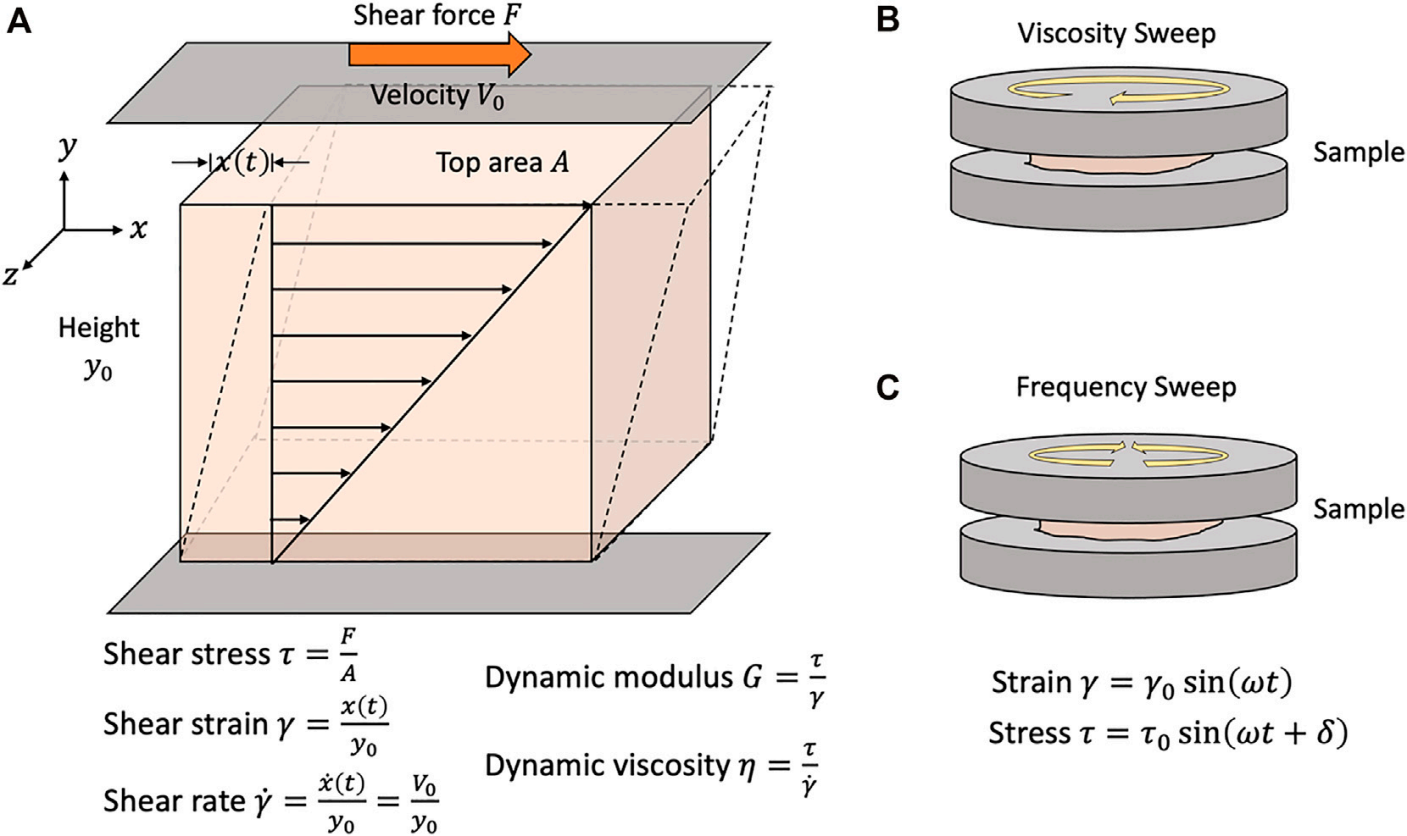
Rheological assessment of biomaterials for bioinks. **(A)** Principles of rheometric analysis. When a shear force is applied to a material, the material is deformed by an amount x(t). The applied shear stress is a function of the applied shear force over the applied area. The shear strain is defined as ratio of the magnitude of this deformation to height of the unit volume. The shear strain rate (or simply shear rate) is time-derivative of the shear strain. Sample material is placed in between two parallel plates, one of which rotates either **(B)** unidirectionally or **(C)** oscillates bidirectionally. Under the no-slip assumption, the sample material is sheared at a rate equivalent to the velocity of the rotating plate V0. The dynamic viscosity of a material is defined as the ratio of the shear stress to the shear rate and is empirically determined by ramping the applied shear rate and measuring the shear stress of the sample. The dynamic modulus of viscoelastic materials is commonly measured while applying oscillatory strain. A maximum strain of $\gamma_{0}$ is applied at a frequency ω . There may be a lag-time between the application of the strain and the measured stress. The phase shift between the stress and strain is given by δ . Viscoelastic materials with predominantly liquid-like characteristics will have a large phase shift, while predominantly solid-like materials will have a smaller phase shift.

The shear storage (G′) and loss (G″) moduli are two measurable indexes representing the elastic and viscous properties of a material, respectively. They can be determined readily using a rheometer. One can sandwich a material sample between two parallel-plates in a rheometer and apply oscillatory shear stress and strain as one of the plates rotates back and forth, as shown in Figure 2. Since the applied strain is oscillatory, the response stress curve will also be oscillatory. The phase shift between these strain and stress curves is defined as the loss tangent (*δ*). There is a larger shift for more viscous-like materials than with more elastic-like materials. Once the loss tangent is known, G′ and G″ can be calculated as the sine and cosine components of the ratio of the maximum shear stress to shear strain.

The storage modulus has been correlated with an improved shape fidelity (Diamantides et al., 2017; Gao et al., 2018; Lee et al., 2020). Storage modulus also correlates with increased polymer concentration (Osidak et al., 2019). A study on gelatin-alginate blends suggested that bioinks with a G′/G″ between 0.25–0.45 produce a good balance between smoothness of the printed filament and structural integrity (Gao et al., 2018). These findings highlight the compromise that must be made. A high storage modulus correlates with increased structural integrity but also decreased extrudability and cell viability. A higher loss modulus correlates with increased uniformity and better shape fidelity, but if the loss modulus is too high, the ink may spread too much upon printing, lowering the shape fidelity.

### Viscosity and Shear-Thinning Property

Another rheometric property used to evaluate whether a bioink is printable or not is its viscosity. To measure viscosity, a sample is placed between two plates in a rheometer, as shown in Figure 2. The sample is subjected to an increasing shear rate when the top plate begins rotates unidirectionally. The shear rate and resultant shear stress is plotted where the slope of the curve is the viscosity. The viscosity quantifies the material’s resistance to deformation by shear. If the slope is constant across the tested range of shear rates, the material is considered Newtonian and has a constant viscosity across a range of shear rates. Otherwise, a material’s measured viscosity is a response to the applied shear force. It is common to plot the apparent viscosity over the shear rate. Newtonian materials will give a constant line. If the viscosity decreases with increasing shear rates, the material is considered a shear-thinning material. If the viscosity increases with increasing shear rates, the material is a shear-thickening material. The rheometric properties and printing parameters of common materials used in 3D bioprinting are available in Table 1.

**TABLE 1:** Commonly used bioinks, their respective properties, and recommended print conditions.

	Viscosity	G′	G”	Cell viability	Cell density	Print moduli	Resolution
Collagen	23.0–43.7 Pa-s	4–2000 Pa	1.4–1000 Pa	>90%	10^6^–10^8^ cells ml^−1^	Extrusion, Laser-assisted	10–100 µm
Gelatin/GelMA	1–5 mPa-s	900–1050 Pa	10 Pa	90–99%	10^6^–10^7^ cells ml^−1^	Extrusion	100–250 µm
Fibrin	3.5–7.5 mPa-s	2.2–2.6 mPa	1.6–2.2 mPa	>85%	10^6^–10^7^ cells ml^−1^	Extrusion	100–200 µm
Pluronic Blends	0.1–500 Pa-s	10,000–16,500 Pa	1,000–4,000 Pa	>80%	10^6^ cells ml^−1^	Extrusion	100 µm
PEG Blends	100–750 Pa-s	500–1,000 Pa	70–110 Pa	>88%	0.5 × 10^6^–10^7^ cells ml^−1^	Extrusion, Drop on Demand	500 µm
Alginate Blends	0.25–200 Pa-s	0.001–1000 Pa	0.1–1000 Pa	80–90%	10^6^–6 × 10^6^ cells ml^−1^	Extrusion	500–1000 µm
Agarose Blends	0.3–10 Pa-s	2–225 Pa	0.7–75 Pa	>85%		Extrusion	300 µm
Hyaluronic Acid Blends	200–10,000 mPa-s	0.1–8000 Pa	1.5–11 Pa	>95%	10^6^–20 × 10^6^ cells ml^−1^	Extrusion, Stereolithography	330–650 µm
Xanthan Gum Blends	2000–3650 mPa-s	850–3000 Pa	100–400 Pa	>90%	1.0–5.0 × 10^6^ cells ml^−1^	Extrusion	200 µm
Cell Dense Bioink Blends	0.7–45 Pa-s	0.75–310 Pa	1–100 Pa	95%	>1.0 × 10^8^ cells ml^−1^	Extrusion, Laser-assisted, Drop-on-Demand	60 µm

Shear-thinning is a desired property of bioinks as it may decrease the chance of clogging. For extrusion-based bioprinting, the bioink will experience shear as it passes through the bioprinter. The shear rate will increase as the extrusion rate increases, but also as the inner flow diameter decreases. A shear-thickening bioink would have an increased apparent viscosity which would make it more likely to clog, therefore making a shear-thinning bioink a better candidate.

Thixotropic bioinks are also favorable bioinks as they also exhibit shear-thinning properties. Whereas traditional shear-thinning materials would exhibit a decreased viscosity as the shear rate is increased, a thixotropic material may exhibit a decreased apparent viscosity as the duration of applied shear is increased, such as through agitation. When the stimulus is removed, the material has a characteristic relaxation time to return to its original viscosity. A study of nanocellulose-alginate blends measured the initial and final viscosities of various blends after an applied shear rate of 1000 s^−1^ for 100 s and demonstrated that a 50% nanocellulose-50% alginate blend produced the best printed shape fidelity which correlated with the highest percent recovery of the original viscosity (Abouzeid et al., 2018).

### Yield Shear Stress

A bioink has a yield shear stress if there is a threshold shear stress that must be overcome before the material deforms. The apparent viscosity of the material before the yield shear stress is infinite as the slope on the shear stress-strain rate curve may appear vertical or very steep.

Several studies have connected the yield shear stress with printability (Mouser et al., 2016; Ribeiro et al., 2018; Kiyotake et al., 2019; Lee et al., 2020). For many bioprints, success relies on being able to build up several layers of a material. To maintain its printed shape, a free-standing structure must be able to support its own weight under gravity, which effectively creates a deforming force proportional to the structure’s mass. Effectively, this means that bioinks with very high apparent viscosities at low shear rates could accomplish the same thing, but this may not be favorable for extrusion.

## Biomaterials Used for Bioink

### Natural Materials

Natural polymers are prominent in 3D bioprinting. Most of these materials are derived from plant or animal sources. Natural polymer hydrogels offer cells with binding sites for their attachment, proliferation, and differentiation. They can be remodeled by cells (Gungor-Ozkerim et al., 2018; Khoeini et al., 2021). The ECM is largely composed of large, fibrillar proteins such as collagen and elastin, as well as smaller glycoproteins, all of which serve as structural and biochemical support for cells in the environment. Several of these proteins have been incorporated into bioinks.

#### Collagen

Collagen is a key structural component of ECM and is found ubiquitously in many tissues (Wang et al., 2020). Of the many subtypes of collagen, collagen type I (COL I) is the most abundant type of collagen. COL 1 hydrogels are frequently used as an *in vitro* 3D cell culture environment (Wang and Ye, 2009; Antoine et al., 2015; Campos et al., 2015; Drzewiecki et al., 2017; Wang et al., 2017; Sobhanian et al., 2019; Moeinzadeh et al., 2021). Collagen is an attractive bioink component. Extraction protocols for COL 1 from animal tissue make use of dilute acids such as acetic acid or hydrochloric acid to produce solutions containing individual collagen fibrils (Rajan et al., 2007). Elevated saline concentrations (>150 mM) and pH (Harris and Reiber, 2007), and temperature (Antoine et al., 2015) promote the reassembly of these fibrils to form collagen hydrogels.

The incorporation of COL 1 into bioinks requires careful planning to ensure that it is compatible with the bioprinting modality. Advantages of incorporating collagen into bioinks include high biocompatibility, increased cell spreading, attachment, proliferation, and more favorable cell behaviors (Stratesteffen et al., 2017). However, COL 1 solutions usually have low viscosities and tend to flow, making 3D shape fidelity difficult to control with solely collagen inks. Low-viscosity bioinks are compatible with droplet-based or laser-assisted bioprinting. It has been tested for fabricating vascularized skin models (Lee et al., 2009; Lee et al., 2010; Lee et al., 2014; Baltazar et al., 2020). To avoid gelation during printing, both the printhead (cold) and build platform (warm) are temperature-controlled. Once deposited, the collagen droplets were misted with a basic solution of sodium bicarbonate to neutralize the pH and promote collagen gelation on a warm buildup platform. To improve its application for extrusion-based bioprinting, higher concentrations of COL 1 have been used to formulate a bioink (Rhee et al., 2016). The use of higher concentrations collagen to formulate bioink increases difficulty in cell mixing and bioionk loading into the bioprinter New methods have been developed to produce more concentrated COL 1 solutions with more favorable properties (Shim et al., 2016; Osidak et al., 2019; Lee et al., 2020). This COL 1, branded Viscoll collagen, is a fractionated collagen solution extracted from animal tendons, and purified using salt precipitation and ion-exchange chromatography (Osidak et al., 2019).

Use of other types of approaches have been attempted for bioprinting of collagen bioinks. For example, aerosol jet printing of COL I and COL II has been shown to result in printed structures with greater stiffness and elastic moduli (Gibney and Ferraris, 2021). This study has report that the mechanical properties of its aerosol jet printed collagen are comparable to dense collagenous tissues and further chemical crosslinking could result in structures that replicate stiff cartilage. For instance, a recombinant COL 3 was used to print a fine film with several hundreds of microns thick for recreating corneal tissues (Gibney et al., 2021).

#### Gelatin and GelMA

Gelatin is a molecular derivative of collagen, created through the irreversible, heat-induced denaturation of collagen proteins (Bello et al., 2020). Gelatin shares a similar molecular structure with collagen. It is often used as a collagen replacement in tissue cultures because of its cost effectiveness. . The use of gelatin in 3D bioprinting enhances cell attachment, decreases average bioink cost, and gives the bioink a reversible gelation property. Although gelatin is a gel at room temperature and lower, gelatin gels melt as the temperature approaches body temperature (37°C). This property makes gelatin a versatile component of many bioinks. Nevertheless, gelatin bioinks without chemical alteration come with fewer functionality than their collagen counterparts (Gopinathan and Noh, 2018).

Gelatin methacryloyl (GelMA) is a gelatin derivative synthesized by the reaction of gelatin with methacryloyl chloride in a phosphate buffer. The reaction induces the presence of methacryloyl substitution groups on the reactive amine and hydroxyl groups of the gelatin amino acid chain (Yue et al., 2015) and makes GelMA amenable to photo-induced chemical crosslinking. GelMA is favored for tissue culture due to its enhanced mechanical and thermal properties when compared to gelatin. UV crosslinked GelMA hydrogels will not melt at physiologically relevant temperatures (∼37°C), whereas gelatin would. GelMA-based hydrogels have been used to culture many cell types, including HUVECs, hMSCs, BMSCs, ADSCs, chondrocytes, and C2C12 cells (Sun et al., 2018). GelMA bioinks were used to print vascularized bone matrix structures using an extrusion-based printer (Leucht et al., 2020). The printed 3D models were employed to confirm paracrine crosstalk between osteogenic and vasculogenic cells in the formation of bone tissues.

#### Fibrin

Fibrinogen is a glycoprotein found in the blood and is a major component of blood clots. Through the action of proteolytic cleavage by the enzyme thrombin, soluble fibrinogen is converted into insoluble fibrin. Fibrin gels share many commonalities with collagen gels. Fibrinogen solutions have a very low viscosity. They are typically mixed with other materials when used for bioprinting to overcome spreading after printing, which will be discussed in the subsequent sections. Fibrin-only hydrogels and bioinks are highly biocompatible, albeit, their mechanical properties are fairly weak (Gopinathan and Noh, 2018). Being that fibrin gels are created through the action of thrombin on fibrinogen, extrusion printing of fibrinogen/thrombin solutions is difficult due to the fact that viscosity increases over time. A high concentration of fibrin (20 mg/ml) bioinks was used for printing 3D microenvironments that promoted human induced pluripootent stem cells (iPSCs) differentiating into mature, electro-physiologically active neurons (Sharma et al., 2020). These neural tissue models closely mimicked spinal cord tissues. In another study, fibrin-hyaluronic acid bioprinted scaffolds showed an ability to support vascular network formation by HUVEC cells through human dermal fibroblast layers (Kreimendahl et al., 2021). This is a stunning finding, demonstrating the dependability of fibrin-based inks when studying vascular tissue.

### Synthetic Materials

While natural polymer materials have excellent biocompatibility, they are difficult to work with both mechanically and chemically (Gopinathan and Noh, 2018). Synthetic materials can be chemically synthesized and tailored for desired properties. For instance, and Pluronics and polyethylene glycol (PEG) can be specialized for improved mechanical properties, printability, crosslinking, water absorption, etc. (Guvendiren and Burdick, 2013). However, synthetic materials usually have poor biocompatibility or poor adhesion sites to support cell attachment, proliferation, and differentiation. Functionalization with adhesion peptides or natural materials will improve their ability to support cell attachment and proliferation.

#### Pluronics

Pluronic F-127 is a thermosensitive hydrogel that provides favorable mechanical properties essential to bioprinting (Diniz et al., 2015). In particular, solutions of 15% (w/v) aqueous Pluronic F-127 or greater undergo a transitions to a gel state from a liquid state at a 20°C (Shriky et al., 2020). This versatile property means Pluronic F-127 gels can be printed at room temperature but liquified at subjecting the print to refrigerator temperatures available in most labs. Moreover, Pluronic F-127 favors cell attachment and collagen deposition (Heilmann et al., 2013). However, one drawback stemming from the use of Pluronics is that they are easily and quickly degraded under physiological conditions. To combat this, researchers frequently crosslink pluronics with hydroxyl groups in order to modify its depsipeptide unit’s chemical structure (Qiu et al., 2011). It has been shown that the hydrogel is a promising option for vascular regeneration in osteoblastic and epithelial tissue (Huang et al., 2006). It was demonstrated that 3D printed GelMA-Pluronic F-127 structures (15% GelMA, 30% Pluronic) are highly printable, easily perfusable, and led to HUVEC cell angiogenesis and vascular branching (Suntornnond et al., 2017). More recently, laser-based forward transfer of 15% (w/v) Pluronic F-127 bioinks has been employed to precisely deposit single cells on a gelatin substrate with predetermined intercell distances of 50, 100, and 200 µm (Zhang et al., 2021). This study demonstrates the precision that can be achieved when using a laser-assisted approach to bioprint Pluronic F-127.

#### Polyethylene Glycol

PEG is another synthetic material widely used in biprinting. PEG can be synthesized through the polymerization of ethylene oxide. It is an attractive material due to its customizable and strong mechanical properties, non-cytotoxicity, and non-immunogenicity. Nonetheless, PEG is biologically inert and must be used in combination with biologically functional materials in order to support cell growth. Polyethylene glycol supplemented hydrogels are commonly used in 3D bioprinting and have been proven to be useful in the study of vascular tissue, bone tissue, and cartilage tissue (Unagolla and Jayasuriya, 2020). Recently, printed cell-laden PEG-gelatin bioinks showed high cell viability and proliferation over time (Piluso et al., 2021). The group’s constructs also showed healthy and well-defined cell morphology, and the subsequent formation of micro-capillary beds. PEG-4MAL bioinks (5% w/v) have also been printed through the drop-on-demand method (Utama et al., 2021). Crosslinked PEG-4MAL hydrogels with a bis-thiol crosslinker and were able to maintain 90% cell viability after 6 days across both PDAC and fibroblast cell lines.

### Cell-Dense Bioinks

Cell-dense bioinks contain a high concentration of cells mixed into the biomaterials. Cell densities in the body are typically much higher than those employed in bioinks, where scaffold/hydrogel material is the primary component. This has been known as matrix- or scaffold-free bioinks. These bioinks rely on self-assembly of cells to form a tissue. Often tissue spheroids are used as building blocks.

One prime example of this is the “Kenzan” method pioneered by Nakayama and his colleagues (Itoh et al., 2015). Pre-formed cell spheroids were robotically impaled onto 160-µm thick microneedles arranged in a regularly spaced grid. The microneedles provided support while the spheroids grew large enough to contact neighboring spheroids and fuse together. Recently, the Kenzan method has been used to fabricate trachea (Taniguchi et al., 2018), cardiac tissues (Arai et al., 2021), and bile ducts (Hamada et al., 2021).

### Sacrificial Bioinks

Sacrificial bioink is referred to those inks which role in the overall bioprint is to provide support or direct the shape of final bioprint. Sacrificial bioinks are used to print structures which are temporary in the overall bioprinting. Employing sacrificial bioinks offers additional dynamic control. Traditional sacrificial materials only persist during the bioprint and for a short span of time into the beginning of the tissue culture before they are removed. For example, gelatin-GelMA systems have been employed to create HUVEC layered channels throughout 3D printed structures (Ouyang et al., 2020). Gelatin was the sacrificial material in this study. The researchers evacuated all gelatin from the 3D printed structure after cellular adherence to the channel walls, resulting in HUVEC-lined vascular mimicry. The same idea has been proposed to use sacrificial cell spheroids (Robu et al., 2019). Sacrificial cell spheroids can grow in the tissue but are connected to a mechanism that could initiate cell death, effectively causing those cells to die off and leave a void space in the bioprint. Employing sacrificial bioinks would give additional dynamic control. Traditional sacrificial materials only persist during the bioprint and for a short-span of time into the beginning of the tissue culture before they are removed. Sacrificial cell spheroids could be allowed to grow and influence the tissue in a certain way before “sacrificing.” Robu et al. demonstrated computer simulations depicting sacrificial cell spheroid structures. The simulations used in the study have shown cells spontaneously relocating within the construct, leading to the formation of a grander, branched structure.

## Strategies Developed for Improving Bioink’s Printability

A wide range of materials have been used as bioinks. However, it is rare that any one material possesses all the properties desired for a specific bioprinting application. For a single material, processing conditions before, during, and after the print can be used to modulate the material’s behavior. Table 2 summarizes processing and formulation strategies used to improve bioink properties for bioprinting. In general, the viscosity of polymeric solutions correlates positively with concentration and negatively with temperature. A good example of this is collagen bioink. Collagen type I (COL1) solutions are typically low-viscosity in nature prior to gelation. A 2014 comprehensive review of bioprinting involving collagen revealed that most studies used concentrations under 10 mg/ml (Antoine et al., 2014). At these concentrations, collagen tends to spread, making it hard to hold a shape or endure a mechanical strength for another layer of collagen deposition during printing. Using a high concentration such as higher than 20 mg/ml to minimize spreading and to improve the spatial accuracy of the bioprint has been attempted (Rhee et al., 2016). However, a higher viscosity in turn makes the collagen harder to process. Another strategy is to bestow additional functionality to the collagen to improve its printability. For instance, methacrylamide functional groups can be added to the collagen to increase its stiffness through crosslinking in the presence of photoinitiators under UV or blue light. Collagen methacrylamide gels formed after UV exposure exhibits increased shear storage moduli when compared to non-modified collagen (Gaudet and Shreiber, 2012), while showing no significant difference in cell viability during printing (Drzewiecki et al., 2017).

**TABLE 2:** Bioink processing and formulation methods that affect overall printability.

Processing method for bioink improvement	Result of implementation	Applicable Bioink(s)
Increase concentration of main ingredient biomaterial	Minimize spreading and improve spatial accuracy	Collagen-based, gelatin-based, agarose blends, alginate blends
Incorporation of methacrylamide functional groups	Increase stiffness through UV crosslinking and increase shear storage moduli	Collagen-based, gelatin-based
Gelatin blending	Improves cell attachment and viscosity, encapsulates bioink components, shape maintenance	Fibrin-based, collagen-based, alginate-based
Hyaluronic acid blending	Allows for alternative crosslinking methods and naturally acts as a signaling molecule for cell migration/proliferation	Collagen-based, gelatin-based, alginate-based, agarose-based
Alginate blending	Temporary structural support to other materials as they are printed/undergo gelation	Collagen-based, fibrin-based
Xanthan gum incorporation	Minimize cell settling and improve shape fidelity	Collagen-based, gelatin-based, fibrin-based
κ-carrageenan incorporation	Viscosity enhancer	Collagen-based, fibrin-based
Gellan gum incorporation	Viscosity enhancer, improve shape fidelity and printing accuracy	Collagen-based, fibrin-based

The third approach is to combine several materials together to form a material blend that exhibits all the desired properties of constituent materials. For instance, we have developed a fibrinogen-gelatin blend for bioprinting cylindrical vascular constructs using an additive lathe-style bioprinter (Freeman et al., 2019). The addition of the gelatin to the fibrinogen enabled the bioink to remain on the cylindrical surface against gravity and increased the viscosity of the overall bioink. All components of the bioink were combined warm so the gelatin would be a solution, and then allowed to cool to encapsulate all the components. The gelatin maintained the shape of the bioprint during enzymatical crosslinking that crosslinked the fibrinogen to form a fibrin fiber network. The placement of the bioprint at 37°C forced it to undergo a significant reduction in volume, suggesting that the gelatin liquified and vacated leaving behind only the resulting fibrin and cells.

Gelatin blending can also be used to improve the biocompatibility of other materials when formulating bioinks. For instance, alginate has excellent printability; however, it does not support cell attachment. Blending gelatin with alginate improves the cell attachment after 3D printing (Jiang et al., 2018).

Hyaluronic acid is another material that is often used in combination with other bioink-forming materials (Levett et al., 2014; Shin et al., 2019; Yu et al., 2021) or chemically modified to allow for alternative crosslinking methods (Skardal et al., 2010). It is a mucopolysaccharide glycoprotein that acts as an important binding and protective agent in human connective tissues. HA naturally acts as a signaling molecule for cell migration and proliferation (Lee et al., 2021), owing to its extensive use in hydrogel formulation. *In situ*-forming, silanized hyaluronic acid hydrogels with tunable mechanical properties were able to sustain cell viabilities of over 80% after 7 days of culture (Flegeau et al., 2020).

Alginate can be combined with other materials as well to increase its stiffness and viscosity for bioprinting. Collagen-alginate blends have been used to print various kinds of tissues (Yang et al., 2018). Alginate salt solutions with sodium or potassium monovalent counterions gel when those monovalent counterions are replaced with divalent ones, such as calcium, magnesium, and barium (Montanucci et al., 2015). Additionally, alginate gelation is reversible if those divalent counterions can be sequestered such as by chelating agents such as EDTA or citric acid. Therefore, the incorporation of alginate into bioinks can also serve to provide a temporary structural support to other polymeric materials as they also undergo gelation. Once the other material has fully gelled, the alginate can be removed by sequestering the divalent cations.

Xanthan gum has also been used to minimize cell settling in bioinks with a lower viscosity (Dubbin et al., 2017) and to improve shape fidelity (Garcia-Cruz et al., 2021). Bioprinting applications do not typically include xanthan in a gel form. Xanthan-based blends (Iijima et al., 2007; Karoyo and Wilson, 2017; Alves et al., 2020) can form gels after annealing at temperature above 40°C followed by cooling, but this particular mechanism has not found an application in 3D bioprinting to date. Like xanthan gum, κ-carrageenan is a viscosity enhancer and has been used to modulate the rheological parameters of other materials (Lim et al., 2020). Carrageenan is a polysaccharide extracted from red seaweed and can only form transient weak gels when annealed in the presence of potassium ions (Hermansson et al., 1991). Recently, carrageenan-based bioinks demonstrated increased interfacial bonding with gelatin-based hydrogels (Li et al., 2018). Gellan gum is a polysaccharide derived from the organism *Sphingomonas elodea* and has been employed as a viscosity enhancer (Melchels et al., 2014). Gellan gum-blends have increased yield stress characteristics, which help improve the printing accuracy and shape fidelity (Mouser et al., 2016). Gellan gum can undergo gelation when cooled from high temperature, which makes it hard to apply gellan gum gelation in biological applications (Graham et al., 2019). New gellan gum chemistries have been explored to improve the processing characteristics of gellan gums (Gering et al., 2021).

Other approaches include the blending of several polymers into a bioink to achieve a better overall printability. For instance, silk fibroin is commonly used in tissue engineering due to its strength and biocompatibility. Direct fibroin gelation requires the use of horseradish peroxidase that is harsh to cells (Kaewprasit et al., 2018). To improve cell survival rate during printing, silk fibroin can be blended with alginate. Alginate protects cells from horseradish peroxidase enzymatic crosslinking and thus improves the cell survival rate during printing. The alginate can be removed by reversing it through the chelation of divalent cations with sodium citrate. The blended bioink shows a better cell viability (Compaan et al., 2017). Silk fibroin has also been printed directly into a suspension bath of PEG/Laponite nanoclay to create a freeform structure (Rodriguez et al., 2018).

Natural polysaccharide polymers have been used frequently to develop various bioink formulations. Due to their biocompatibility, polysaccharides are commonly used as cell encapsulants for delivering cell therapies. Mammalian cells, however, have limited abilities to interact with polysaccharides without chemical modifications. Several plant-based polysaccharides have been tested for bioprinting. For instance, Agarose is a naturally occurring polysaccharide that is generally marine red algae. Concentrations as low as 0.13% (w/v) agarose are able to form stable gels (Ichinose and Ura, 2020). It is, however, difficult to directly incorporate agarose with cells, as it must be heated to supraphysiological temperatures in order to form gel after cooling. To overcome this, agarose can be used with other materials. For instance, polycaprolactone (PCL) can be mixed with agarose to formulate a bioink. A 2% (w/v) PCL-agarose bioink has been shown effective for printing mesenchymal stem cells (MSCs) into hyaline cartilage tissues with 80% cell viability (Daly et al., 2016). Chemical modifications can also be used to improve agarose’s printability by introducing additional functional groups. Carboxylated agarose (40% carboxylation with carboxylic acid groups on the polysaccharide backbone) yields free-standing complex structures with high levels of post-print layer adhesion, ensuring that the structures do not collapse under their own weight (Gu et al., 2020). The bioinks in this study were supplemented with human nasal chondrocytes and showed typical morphological profiles and mitotic cell division after printing. The integrin-binding peptide RGDSP improves cell attachment. Stiff agarose-based bioinks more efficiently promoted chondrogenesis (Arya et al., 2019).

Another plant-based biomaterial that is used widely for formulating bioinks is alginate. Alginic acids are extracted from brown algae and composed of α-L-guluronic acid and β-d-mannuronic acid residues linked by glycosidic linkages (Venkatesan et al., 2015). Several viscosities of alginic are available, depending on the average molecular weight. The various viscosities make alginate a versatile biomaterial. It does not provoke much of an inflammatory response when implanted *in vivo*, it can entrap water and allow it to diffuse from the inside out, and can support the growth of multiple cell types (Gopinathan and Noh, 2018). Alginate-based bioinks have been shown suitable for nerve tissue engineering and were demonstrated as scaffolds for repairing damaged spinal cord tissue (Grijalvo et al., 2019). Low-concentration/low-viscosity (0.2–1.0%w/v) alginate hydrogels proved favorable for bioprinting Schwann cells with improved cell viability and function (Ning et al., 2019).

Chitosan, a deacetylated chitin from shrimp shells, is a commonly used as dietary supplements for weight loss through gastrointestinal fat binding (Ni Mhurchu et al., 2004). It is considered one of the most ‘tunable’ biomaterials, as the facile chemical modifications of its polysaccharide chain will bestow it a wide range of optimizable biological profiles (Kean and Thanou, 2010). This structural variability makes it a fine candidate for bioprinting. It was reported that a tissue structure printed using chitosan chitoclear-raffinose pentahydrate bioinks can remain mechanically strong after 80% dehydrated (Bergonzi et al., 2019).

## Machine Learning (ML)-Enabled Bioprinting Optimization

Bioprinting involves many components and various steps. The prediction of the outcome of bioprinting becomes extremely challenging without a workable computational model; thus, the optimization has been heavily dependent upon trial-and-error. Such empirical approaches can be emulated by machine learning or ML. ML has been specifically used for *in situ* printing, i.e., depositing bioinks directly on a target surface to ensure geometrical accuracy and compensating for moving and various topographies of surfaces (e.g., wound sites, cardiac tissue, etc.) (Zhang et al., 2021; Zhu et al., 2021). *In situ* printing is autonomously performed via a robotic arm or a handheld printer, which is advantageous for surgical application considering the minimal weight and size of the machine (Zhang et al., 2021). Given the nature of *in situ* printing, introducing ML can provide open-loop, closed-loop, and predictive feedback-control systems to assist with design and printing optimization (Zhu et al., 2021).

ML is a new paradigm for bioprinting. Although still in its infancy, ML has been used to address several key aspects of bioprinting. ML models use computer algorithms to replace human data collection/analysis (input) and determine a correlation (output) (An et al., 2021). Stemming from AI, ML algorithms can learn and improve by themselves. There are five main methods of ML: supervised, unsupervised, reinforcement, semi-supervised, and deep learning (An et al., 2021; Shin et al., 2022). Supervised relies on the labelled input data (typically taken from sensors or databases), which is used as training data (Shin et al., 2022). In unsupervised ML, the algorithms discover a pattern or relationship between unlabeled input data to determine an output (An et al., 2021; Shin et al., 2022). As the abundance of data increases, supervised and unsupervised ML becomes more feasible at optimizing bioink and bioprinting (An et al., 2021; Shin et al., 2022). Semi-supervised ML combines the idea of supervised and unsupervised. A small amount of labelled data and a large amount of unlabeled data is present (Shin et al., 2022). Reinforcement ML finds the functions between the input and output, similar to supervised ML, but is based on a reward system algorithm called environment. The ML learns from the environment rewarding consequences of trial-and-error of different states. The environment is repeated to obtain as many rewards as possible to obtain a desired state (An et al., 2021; Shin et al., 2022). Deep learning ML uses algorithms that learn independently and automatically. It is best applied when variables are not understood during data processing and large data sets that require extensive computational time are used. Deep learning ensures performance without processing the variables by constantly applying algorithms to new datasets (An et al., 2021; Shin et al., 2022).

Bioprinting proceeds in three processing steps: pre-printing, printing, and post-printing. Pre-printing pertains to the printing parameters, bioink selection, construct design, and environment setup that are essential to having an optimized deposition. ML can be applied here by incorporating data to determine the best printing conditions prior to printing (Shin et al., 2022). This data can be collected from Big Data, a source of data on histology, tissue geometries, molecular profiles, immunochemistry, and clinical imaging of tissues and organs. The information in Big Data on bioprinting is limited (An et al., 2021). The concept of creating digital twins of human organs entails creating a tissue map of a human organ using advanced imaging and 3D reconstruction (An et al., 2021). The exploration of digital twins has just begun, but if widely used it can provide an effective model of cell and tissue properties for anatomically accurate bioprints. Adding more information collected by ML into Big Data and utilizing digital twins of human organs can greatly assist in improving printing quality and accelerating printing time. During the actual printing process, ML can analyze the construct as it is being printed and use algorithms to adjust parameters in real-time. The post-printing process determines the quality of printed constructs and is crucial for improving future prints (3).

The integration of machine learning in each of these steps can assist in complex printing, shape fidelity, printing optimization, and tissue regeneration. The following sections will describe pre-printing, printing, and post-printing innovations that use AI, specifically ML, to advance 3D bioprinting.

### Printing Process

#### Closed-Loop AI Printing

Rather than relying on previous data and using AI pre-printing, closed-loop AI uses sensors like cameras and strain gauges to record real-time (online) data during the printing process. This data is collected and run through ML algorithms to identify printing defects, give feedback to the printer, and correct printing errors for improved printing quality and precision (Zhang et al., 2021; Zhu et al., 2021).

Disruptions to printing dispensing such as temperature, air pressure, and bioink viscosity changes can greatly affect the droplet behavior in drop-on-demand (DOD) bioprinting (Shi et al., 2019; Zhu et al., 2021). Drop-on-demand bioprinters dispense microvolumes of a low-viscosity cell suspension. The satellite droplets are frequently an issue. Ideally, DOD bioprinting should precisely deposit cell-laden drops in specified locations, but unoptimized printing process sprays satellite droplets in unwanted locations (Shi et al., 2018). One method optimize DOD printing is to use a voting-based law to adjust the piezoelectric voltage of the inkjet printer based on images collected during printing. The images can be used to note the number of satellites, volume of the droplet, and extrusion speed. This data is then processed by a classification algorithm, which produces a histogram that plots the voting scores and the corresponding voltages. From this, the optimal voltage can be obtained and applied in the corresponding prints (Zhu et al., 2021).

**FIGURE 3:**
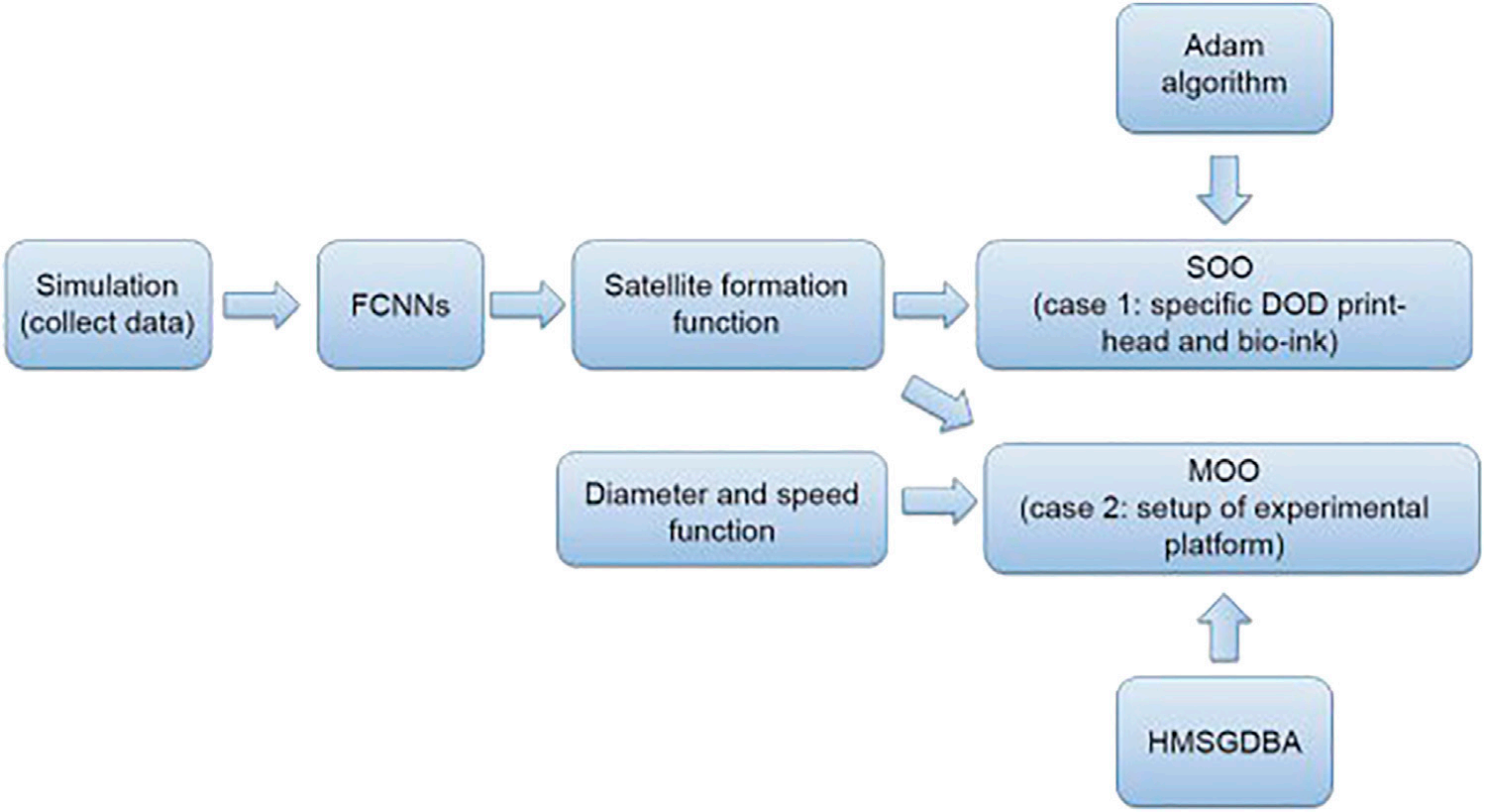
Flowchart depicting schematics of multi-objective optimization design for improved drop-on-demand bioprinting (Shi et al., 2019).

Another application of real-time closed loop control to ensure printing quality in DOD is the use of a multilayer perceptron (MLP) learning model. The MLP was applied to minimize the formation of satellite droplets for a low-viscosity cell-laden suspension (Shi et al., 2018). The MLP was informed by a computational fluid dynamics (CFD) simulation, which was first validated and demonstrated a high fidelity to actual high-speed camera footage of droplet dispensing. The trained MLP model achieved a predictive accuracy of 90% on a range of liquid viscosities between 1–10 cP. The feasibility of using CFD simulations for printing more complex multi-component bioink decreases, due partially to poor understanding of exact forces governed during the printing. Diminished printing precision and pattern accuracy come from droplet satellites and slow printing speeds. Typically, at slower speeds droplets will be overly deposited and create inaccurate structures, which can cause issues with cell viability and overall cell arrays. To overcome these problems, a multi-objective optimization (MOO) design through ML for piezoelectric drop-on-demand printing has been explored. Piezoelectric drop-on-demand printing releases droplets based on mechanical pulse stimulations applied to the bioink in the printhead. In this scenario, three different types of cell-laden bioinks were tested based on varying viscosities from differences in concentrations of HeLa cells and sodium alginate (SA). During printing, a camera was used to record the droplet formation and trajectory for analysis (Shi et al., 2018). Furthermore, fully connected neural networks (FCNNs) were used to interrogate satellite formation and corresponding printing parameters as a preliminary for MOO (Shi et al., 2019). This innovation also incorporated a hybrid multi-subgradient descent bundle method with an adaptive learning rate algorithm to combine the multiple sub-gradient descent bundle method and Adam algorithm to ensure calculation stability and accomplish an effective MOO method that optimizes piezoelectric drop-on-demand printing (Figure 3) (Shi et al., 2018). This advanced multi-component system effectively created droplets with no satellites, smaller droplet diameter, and drop-on-demand printing at a faster rate than commercial drop-on-demand methods. The voltages used in the piezoelectric drop-on-demand printing were optimized to print primary droplets (drops containing no satellites), which allows for controllable cell deposition (Shi et al., 2019). By improving printing precision, more reliable cell arrays can be created and at a smaller scale for vaster applications. Also, due to the complexity of this approach, it may be hard to replicate these experiments in other labs, which will make advancements on this specific design more difficult.

Bioprinting involves many steps. Even with highly optimized printing parameters, errors or failures may still occur. A failure can be detected by the operator who can correct the errors. However, this might not be ideal when automation or massive production is desired. Printing error can arise from individual layer defects and is difficult to notice post-printing due to the homogeneity of the printed construct. Rather, analyzing printed layers using 2D images in a closed-loop feedback system can provide insight on intrinsic defects (Zhu et al., 2021). A multilayer convolutional neural network (CNN) was developed to autonomously detect anomalies and irregularities of bioprinting (Jin et al., 2021). The model is able to detect different defects common to bioprinting such as irregular, non-uniform or discontinuous printed lines for layer infill patterns of various geometries based on image analysis (Jin et al., 2021; Zhu et al., 2021). It can be used to detect errors and halt the printing if necessary (Jin et al., 2021). In the future, other ML-based approaches could provide ways to salvage prints.

Shape fidelity can also be maintained by collecting a series of data from printed constructs and using their information to optimize a target shape. One promising approach utilized a hierarchical machine learning (HML) approach to optimize shape fidelity error under 10% for a Freeform Reversible Embedding of Suspended Hydrogels (FRESH) bioprinting method (Bone et al., 2020). The FRESH bioprinting prints hydrogel bioinks into a support bath also consisting of a hydrogel, and the physical interactions between the separate materials is key to printing. The HML approach demonstrated the feasibility of using smaller datasets for optimization. Furthermore, the model’s middle layer was incorporated with known physical relationships governing viscosity, shear rate, pressure, and printed linewidth. Even without knowledge of how these parameters interact with each other, the influence of these parameters was weighted up or down based on their correlations with the response variables using the least absolute shrinkage and selection operator (LASSO). In practice, the HML approach could help choose the optimal processing parameters for any given bioink.

### Post-Printing Process

Printing parameters like geometric accuracy and resolution are contingent on bioprinter settings and the type of bioink used. After constructs are printed, there is no standard method to evaluate and compare them either. Optimizing parameters are essential to ensure cell viability, structural integrity, and tissue replication, but involve tedious trial-and-error testing. Research was conducted in an effort to assess and output optimal parameters in a timely fashion and further advance bioprinting. The parameter optimization index (POI) was developed, where the best POI maximized accuracy and minimized theoretical shear stress (TSS) given by (Webb and Doyle, 2017):

$$POI=Accuracy\cdot \frac{1}{TSS}$$ (1)

The following POI values were also evaluated: POI for the same bioink in multiple experiments

$$POI=\frac{1}{t_{line}\;\cdot \;D_{G\;}\cdot \;p}$$ (2)

$where\;D_{G}=nozzle\;gauge,\;t_{line}=line\;thickness,\;p=pressure$ (Webb and Doyle, 2017) and the POI for the specific bioink with n number of parameters, normalized to the max POI (Webb and Doyle, 2017):

$$POI_{i}=\frac{POI_{i}}{POI_{MAX,n}}$$ (3)

These equations were extensively tested with 72 different configurations with the Inkredible + bioprinter (Webb and Doyle, 2017). The bioink was composed of 7% alginate and 8% gelatin, and the printing parameters varied using 25-, 27-, and 30-gauge print nozzles, 1–6 mm/s print speeds, and 100–250 kPa pressures. Successfully, using this POI gives a valuable assessment of bioinks and printing parameters. This is a promising approach to optimizing printability; however, temperature is assumed constant throughout the printing process, and the POI method is only applicable for straight nozzles (constant TSS), not conical nozzles (variable TSS) (Webb and Doyle, 2017). Since temperature can greatly influence other parameters and cell viability and flexibility in printing nozzles is better, it is necessary to address these limitations to improve the versatility of the POI. Future steps in this research should also be taken to create more complex and multilayer printing patterns, considering only a single layer zig-zag line was analyzed (Webb and Doyle, 2017). Because it aims to minimize shear stress on the bioink while achieving highly geometrically accurate structures—ideal for ensuring cell proliferation and replicating anatomical structures—an application in tissue modeling and drug testing is close in proximity.

The printing process involves many steps and materials including cells. More often one can easily acquire qualitative, but not quantitative data from bioprinting. ML can be a powerful tool to circumvent these difficulties. The Bayesian Optimizer developed by Ruberu, et al. is a combination of ML and hardware for optimizing extrusion printing (Ruberu et al., 2021). It uses ML to quantify printability—including filament formation and layer deposition—via a print scoring system ( Print score=|Layer Stacking Score|+|Filament Morphology Score| ) that assesses filament morphology and pore structure through layer stacking. A negative score indicates under-gelation/droplet, a positive score means over-gelation/no filament, and a score equal to zero represents the ideal bioink. This model offers a new approach to optimize printing parameters with the least amount of experiments. The print score is completed based on photographs of the constructs and the researcher’s best judgement. The algorithm will then produce a probabilistic model that suggests the next printing parameters. This cycle continues with input of the next experiment’s print score and production of new printer settings until an ideal score/construct is reached, indicating that the printer has optimized the constructs parameters. In this case, a ranging composition of GelMA and hyaluronic acid methacrylate bioinks were printed using the EnvisionTEC 3D Bioplotter. The Bayesian Optimization algorithm successfully diminished the experimental process time in comparison to trial-and-error parameter experimentation, and it can be applied to other bioinks that are used in extrusion based bioprinters. The results depict a successful application of ML in bioprinting, with the primary issue being that scoring is done based on photographs. Evidently, this can create bias in experimentation, and flaws the experiments. In the future, it is worth considering working towards an automatic point scoring system.

Bayesian optimization ML has also been applied to predict critical bioprinting parameters from experimentally collected data and minimize the number of iterative experiments necessary to formulate desired bioinks. The Bayesian optimization ML was used along to optimize gelatin methacryloyl and hyaluronic acid methacrylate bioinks under 50 iterative experiments (Ruberu et al., 2021). A classification-based ML was used to strengthen a bioink blend of atelocollagen, fibrin, and hyaluronic acid (Lee et al., 2020). To quantify the success of printing, several combinations of the three biomaterials were printed and classified by whether the bioink clogged the printing nozzle and how closely the printed shape matched the intended shape. Rheological measurements such as the viscosity and the shear storage and loss moduli were measured, as these are commonly measured in order to empirically assess bioinks. Their ML approach determined that a yield shear stress << 10^3^ Pa was required to avoid the clogging of the printer nozzle, while a shear storage modulus >> 10^3^ Pa for well-defined extruded bioink filaments closely matching the desired printed design. Other groups have also experimentally linked yield shear stress as an important rheological parameter for printed shape fidelity (Ribeiro et al., 2018; Kiyotake et al., 2019).

## Current Bioprinting Limitations and Advancements

Despite the novelty and importance of bioprinting in tissue engineering, optimization is a prominent complication in successfully printing bioinks. Printing parameters like nozzle temperature, printing speed, extrusion or pneumatic pressure on bioinks, print-bed temperature, nozzle gauge/diameter, cell viability, etc. are factors that have to be optimized for every bioink. For instance, gelation temperature of a GelMA and COL 1 bioink fluctuates based on their composition (Stratesteffen et al., 2017). Having a homogeneous bioink can warrant more controls over biological and geometric properties of the final printed tissue constructs (Bhattacharyya et al., 2021). Furthermore, extrusion and pneumatic-based printheads require a certain pressure depending on viscosity and composition of the bioink; but a high shear stress will compromise the cell viability. Finding an optimal pressure that promote cell proliferation is tedious and difficult. It also affects other printing parameters such as temperature and nozzle diameter (Webb and Doyle, 2017). Creating an ideal bioink and ultimately a stable structure while maintaining the cell viability are contingent on the balance of these aggregate parameters. Not only is this equilibrium difficult to achieve—making bioprinting optimization burdensome and mostly reliant on trial-and-error—but also there are no prevalent methods for quantitatively analyzing printability post-printing (Webb and Doyle, 2017; Ruberu et al., 2021). These need to be resolved in order to fully realize the power of the bioprinting.

High resolution is an ultimate goal of post-fabrication because it opens the possibility for biofabricating smaller scale tissue engineered structures, complex geometries, and more precisely *in vivo*-mimetic tissues. Combining machine learning with 3D bioprinting has allowed for accelerated fabrication and optimization of bioinks and printing parameters, even on geometrically irregular surfaces. By understanding current, predicting future, and preparing for inconsistent states, ML has reduced printing errors. These advances have been demonstrated *in situ*, which potentiates on-the-spot treatment of patients where time and precision determine their survival (i.e., car accidents, ambulances, emergency surgeries). Despite advancements that have allowed filaments to reach 100 μm, many structures like scaffolds require even more precision (Tashman et al., 2021). Resolution also becomes essential when creating vasculature via 3D printing. Printing fully functional vascular networks at a 3D, detailed scale is still under development. Complications in this process consist of mimicking topology and maintaining continuous perfusion (Cadle et al., 2021). Pluronic F-127 as a sacrificial ink has been significantly studied, leading to milestone thick vascularized tissue models (Kolesky et al., 2016), and with high cell density (Skylar-Scott et al., 2019). The technique was applied to create a perfusable kidney model with intact epithelial lumen, approximating the proximal tubule system (Homan et al., 2016). A subsequent vascularized tissue model produced mature, polarized epithelial cells from pre-tubular aggregates (Homan et al., 2019). Resolution also concurs with printing precision, which is essential for scaffold construction and cell seeding post-fabrication and for creating cell arrays with drop-on-demand printing (Aguilar et al., 2019; Shi et al., 2019; Fortunato et al., 2021). Precision pertains to bioink fabrication, filament placement/deposition, and cell loading. In the case of drop-on-demand printing, a bioprinting technique of emitting droplets for potential encapsulation or creating a cell array, lower droplet/printing speeds result in over deposition of droplets and therefore droplet structural issues and cell array inconsistencies. Lower droplet speeds are often used to compensate for other issues like imperfect bioink viscosity and inaccurate droplet placement. This becomes further complicated when introducing cells to the droplet because incorrect placement can cause inconsistencies in cell array results and potentially risk cell viability if nutrients are unable to reach the cells (Shi et al., 2019).

## Concluding Remarks

Current limitations in 3D bioprinting entail achieving a higher printing resolution, homogeneous bioink (especially when incorporating cells), and optimized scaffold structure. Numerous software and hardware technological advancements have contributed to diminishing these constraints. However, despite current bioink and bioprinter optimization developments, bioprinting has a long way to go before it is deemed as perfect.

Natural polymers remain the primary choice for bioink formulation. Despite the processing limitations they present, several promising approaches and chemical modifications have already demonstrated how these limitations can be overcome. Collagen is a prime example. Several collagen-based bioinks have overcome collagen’s inherently low viscosity, shape fidelity, and low gel stiffness by incorporating polysaccharide such as alginate, xanthan, gellan gum, and agarose. Low-viscosity is also no longer necessarily a limitation as demonstrated by the use of support baths such as the FRESH technique. Already other large polymeric ECM proteins such as elastin are being explored as potentially promising bioink components, and the use of similar approaches will be important to facilitate elastin’s prominence in bioprinting-based biofabrication.

Errors and variability during bioprinting experiments are still a limiting factor. It is apparent from process optimization experiments that even under the same operating conditions printing failures can still occur. Although still in its infancy, the incorporation of ML and computer-aided approaches may serve to minimize the occurrence of processing errors, or even enable ways of dynamically handling errors when they occur. ML also can leverage existing datasets of biomaterial research and 3D cell culture to better inform bioprinting for tissue engineering.
